# Supplementary material for: Characterization of interactions’ persistence in time-varying networks
Source: Sci Rep. 2023 Jan 14;13:765. doi: 10.1038/s41598-022-25907-7 (PMC9840642; doi:10.1038/s41598-022-25907-7)
Supplement: Supplementary file 1 — Supplementary Information. [file 41598_2022_25907_MOESM1_ESM.pdf]

Supplementary Materials for the manuscript entitled: Characterization of interactions' persistence in time-varying networks

Francisco Bauzá Mingueza, Mario Floría, Jesús Gómez-Gardeñes, Alex Arenas, & Alessio Cardillo

Supplementary Note S1: Approximation for the average of a function of random variables

Here, we comment about the conditions for which the approximation of Eq. (6) of the main manuscript is fulfilled. For this purpose, we rewrite the term $\frac{|\cap|}{2\mathcal{L}-|\cap|}$ as a function of random variables

$$\frac{|\cap|}{2\mathcal{L}-|\cap|} \equiv \frac{x}{y-x}\,. \tag{S1}$$

Then, we perform the Taylor expansion of such a function around the point $\big(\langle x\rangle, \langle y\rangle\big)$:

$$\begin{aligned}\frac{x}{y-x} \approx \frac{\langle x\rangle}{\langle y\rangle-\langle x\rangle} + \sum_{k=1}^{\infty}\frac{1}{k!}\left[\left(\frac{\partial^k f(x,y)}{\partial x^k}\right)_{(\langle x\rangle,\langle y\rangle)}\big(x-\langle x\rangle\big)^k + \left(\frac{\partial^k f(x,y)}{\partial y^k}\right)_{(\langle x\rangle,\langle y\rangle)}\big(y-\langle y\rangle\big)^k\right] + \\ + \sum_{n=1}^{\infty}\sum_{m=1}^{\infty}\frac{1}{n!\,m!}\left(\frac{\partial^{m+n} f(x,y)}{\partial x^n\partial y^m}\right)_{(\langle x\rangle,\langle y\rangle)}\big(x-\langle x\rangle\big)^n\big(y-\langle y\rangle\big)^m\,,\end{aligned}$$

which, by replacing the expression of the derivatives, yields:

$$\begin{aligned}\frac{x}{y-x} \approx \frac{\langle x\rangle}{\langle y\rangle-\langle x\rangle} + \sum_{k=1}^{\infty}\left[\frac{\langle y\rangle}{(\langle y\rangle-\langle x\rangle)^{k+1}}\big(x-\langle x\rangle\big)^k + \frac{(-1)^k\langle x\rangle}{(\langle y\rangle-\langle x\rangle)^{k+1}}\big(y-\langle y\rangle\big)^k\right] + \\ + \sum_{n=1}^{\infty}\sum_{m=1}^{\infty}\frac{(-1)^k}{m!}\,\frac{C_x\langle x\rangle + C_y\langle y\rangle}{(\langle y\rangle-\langle x\rangle)^{n+m+1}}\big(x-\langle x\rangle\big)^n\big(y-\langle y\rangle\big)^m\,,\end{aligned} \tag{S2}$$

where $C_x = \frac{(n+m-1)!}{(m-1)!}$ and $C_y = \sum_{i=1}^{m}\frac{(n+m)!(m+i-2)(m+i-1)}{(m-1)!}$.

Given the above expression, we apply the average operator to both the function and the series. In such a case, the first-order terms vanish since $\Big\langle\big(x-\langle x\rangle\big)\Big\rangle = \Big\langle\big(y-\langle y\rangle\big)\Big\rangle = 0$ and, therefore, only the higher order terms remain. Replacing the expression of the partial derivatives, we get:

$$\begin{aligned}\left\langle\frac{x}{y-x}\right\rangle \approx \frac{\langle x\rangle}{\langle y\rangle-\langle x\rangle} + \sum_{k=2}^{\infty}\left[\frac{\langle y\rangle}{(\langle y\rangle-\langle x\rangle)^{k+1}}\Big\langle\big(x-\langle x\rangle\big)^k\Big\rangle + \frac{(-1)^k\langle x\rangle}{(\langle y\rangle-\langle x\rangle)^{k+1}}\Big\langle\big(y-\langle y\rangle\big)^k\Big\rangle\right] \\ + \sum_{n=1}^{\infty}\sum_{m=1}^{\infty}\frac{(-1)^k}{m!}\,\frac{C_x\langle x\rangle + C_y\langle y\rangle}{(\langle y\rangle-\langle x\rangle)^{n+m+1}}\Big\langle\big(x-\langle x\rangle\big)^n\big(y-\langle y\rangle\big)^m\Big\rangle\,.\end{aligned} \tag{S3}$$

According to the above expression, we are entitled to say that $\left\langle\frac{|\cap|}{2\mathcal{L}-|\cap|}\right\rangle \approx \frac{\langle|\cap|\rangle}{2\langle\mathcal{L}\rangle-\langle|\cap|\rangle}$ only when the higher order moments of the marginal and joint probability distributions of both variables are negligible.

Supplementary Note S2: Analytical estimation of the average temporality: the case of the activity driven model

Here, we present an extension of the theoretical estimation of the average temporality, $\overline{\mathcal{T}}$, where we assume that the process governing the formation of the time-varying interactions is the activity driven model (henceforth indicated as ADM) introduced by Perra *et al.* [1]. The ADM, is a well-known model for the generation of time-varying networks. The model is based on a vertex property called *activity*, a_i, encoding the probability that, at each time, a vertex i activates and generates m interactions (edges) towards other vertices (either active or not) in the network.

Assuming that the ADM rules the existence of interactions among vertices, we derive an expression for the probability that the edge between vertices i and j, x_{ij} , exists. Assuming that the activity of a vertex, a_i, and the number of edges generated by each active node, m, do not change across time; we can rewrite the probability that an edge exists between vertices i and j, x_{ij}, [see Eq. (7) of the main manuscript] as:

$$x_{ij} = a_i \frac{m}{N-1} + a_j \frac{m}{N-1}\left(1 - a_i \frac{m}{N-1}\right) \approx \left(a_i + a_j\right)\frac{m}{N-1}\,, \tag{S4}$$

where N is the number of vertices in the network, and the approximation holds true for $a_i \frac{m}{N-1} \ll 1$. Using the above result, the expression for the estimated average temporality, $\overline{\mathcal{T}}_{\text{th}}$, can be written as:

$$\begin{aligned}\overline{\mathcal{T}}_{\text{th}} &= 1 - \frac{\langle x_{ij}^2\rangle}{2\,\langle x_{ij}\rangle - \langle x_{ij}^2\rangle} = 1 - \frac{\langle a_i^2 + a_j^2 + 2a_i\,a_j\rangle}{2\,\frac{N-1}{m}\,\langle a_i + a_j\rangle - \langle a_i^2 + a_j^2 + 2a_i\,a_j\rangle} = \\ &= 1 - \frac{\langle a^2\rangle + \langle a\rangle^2}{2\,\frac{N-1}{m}\,\langle a\rangle - (\,\langle a^2\rangle + \langle a\rangle^2)}\,.\end{aligned} \tag{S5}$$

In particular, to get the above expression we assume that the activity distribution is the same for all nodes (*i.e.*, $\langle a_i^n\rangle = \langle a_j^n\rangle \equiv \langle a^n\rangle$), and that the activities are independent from each other $\langle a_i\,a_j\rangle = \langle a_i\rangle\langle a_j\rangle$.

According to Eq. (S5), it is possible to estimate $\overline{\mathcal{T}}_{\text{th}}$ by just computing the first two moments of the activity distribution, $P(a)$. To estimate the values of activity a_i directly from the data, one can use the method presented by Perra *et al.* in [1] (and, more specifically, Eq. (14) from its Supplementary Material).

Supplementary Note S3: Analysis of synthetic time-varying networks

In this section we characterise synthetic time-varying networks used to study the effects induced by the presence of a fixed/mutating core of interactions on the average temporality, $\overline{\mathcal{T}}$. We introduce first the algorithms used to generate networks with different core's types and, then, we discuss how these structures affect the temporality.

Supplementary Note S3.1: Algorithms to generate synthetic networks with time-varying cores

We present here two simple algorithms to generate synthetic time-varying networks with a set of persistent interactions (*i.e.*, its *core*). These algorithms allow to control the level of edges persistence (*i.e.*, the average temporality, $\overline{\mathcal{T}}$) of the corresponding networks as well as the origin of such a persistence. In both cases, a certain number of edges appear in consecutive snapshots. In one case, the aforementioned edges are the same for all the snapshots, whereas in the other case, the persistent edges change across pairs of adjacent snapshots. Both algorithms consist in the generation of an undirected and unweighted network for each snapshot having fixed numbers of nodes and edges. The input parameters of the algorithms, which do not change through the whole generation process, are the numbers of: snapshots N_s, nodes N, edges, $\mathcal{L} \in \left[0, \frac{N\,(N-1)}{2}\right]$; and the percentage of edges from one snapshots which appear also in the next one, $\kappa \in [0, 1]$. In both algorithms, we begin by generating the network corresponding to the first snapshot which constitutes our "seed." Then, we generate the other snapshots controlling their edges' persistence.

In the first algorithm, which we call the FIXED CORE method (see Algorithm 1), we select uniformly at random $\mathcal{L}' = \kappa\,\mathcal{L}$ edges out of the $\mathcal{L}$ existing in the first snapshot. These $\mathcal{L}'$ edges will exist in all the snapshots, and constitute the *fixed* core of the interactions, whereas the remaining $(1 - \kappa)\,\mathcal{L}$ edges existing in each snapshot are generated according to the generating method used to create the seed. In the second algorithm, called the MUTATING CORE method (see Algorithm 2), for each snapshot i we select uniformly at random $\mathcal{L}' = \kappa\,\mathcal{L}$ edges

among those of snapshot $i-1$, and $(1-\kappa)\,\mathcal{L}$ edges are generated according to the generating method used to create the seed. We considered as seed network, G_{seed}, the Erdős-Rényi graph generated via the $G(N,\mathcal{L})$ method [2].

Algorithm 1: FIXED CORE method

```
Input: Number of nodes, N; number of edges, L; number of snapshots, N_s; percentage of copied
       edges, κ; seed network, G_seed.
Output: Dataset[] // Set of N_s graphs

L' = κL // number of edges in the core
Dataset[0] = G_seed // graph with N nodes and L edges
FixedCore = ∅ // set of L' edges

while size(FixedCore) < L' do
    Select uniformly at random one edge e from G_seed;
    Add e to FixedCore;

i = 1 // set counter

while i < N_s do
    G = ∅ // empty graph
    G = FixedCore;
    while size(G) < L do
        Add edge to G // according to the G_seed generating method
    Dataset[i] = G;
    i = i + 1;

return Dataset
```

Algorithm 2: MUTATING CORE method

Input: Number of nodes, N; number of edges, $\mathcal{L}$; number of snapshots, N_s; percentage of copied edges, κ; seed network, G_{seed}.

Output: Dataset[ ] `// Set of` N_s `graphs`

```
L' = κL // number of edges in the core
Dataset[0] = G_seed // graph with N nodes and L edges

i = 1 // set counter

while i < N_s do
    G = ∅ // empty graph
    while size(G) < L' do
        Select uniformly at random one edge e from Dataset[i − 1];
        Add e to G;
    while size(G) < L do
        Add edge to G // according to the G_seed generating method
    Dataset[i] = G;
    i = i + 1;
return Dataset
```

To have a more versatile control on the origin of the edges' persistence, we devised another algorithm (see Algorithm 3) which interpolates between the FIXED CORE and the MUTATING CORE ones. This algorithm requires an additional parameter $\alpha \in [0, 1]$ regulating how many of the $\mathcal{L}'$ persistent edges belongs to the fixed core and, consequently, how many of them we have to extract from the previous snapshot. The algorithm works as follows: after generating a seed network, G_{seed}, we extract from it uniformly at random $\mathcal{L}'' = \alpha\,\kappa\,\mathcal{L}$ edges which will form part of the fixed core of all the snapshots. Then, for each snapshot i we select uniformly at random $(1-\alpha)\kappa\,\mathcal{L}$ edges from snapshot $i-1$. Finally, we add the remaining $(1-\kappa)\,\mathcal{L}$ edges according to the method used to generate G_{seed}. It is worth to note that $\alpha = 0$ or $\alpha = 1$ correspond to the MUTATING CORE and FIXED CORE algorithms, respectively.

Algorithm 3: Interpolating method

Input: Number of nodes, N; number of edges, $\mathcal{L}$; number of snapshots, N_s; percentage of copied edges, κ; seed network, G_{seed}; parameter; α.
Output: Dataset[ ] `// Set of` N_s `graphs`

$\mathcal{L}' = \kappa\mathcal{L}$ `// number of edges in the core`
$\mathcal{L}'' = \alpha\,\kappa\,\mathcal{L}$ `// number of fixed edges in the core (fixed core)`
Dataset[0] $= G_{\text{seed}}$ `// graph with` N `nodes and` $\mathcal{L}$ `edges`
FixedCore $= \emptyset$ `// set of` $\mathcal{L}'$ `edges`

while *size(FixedCore)* $<\ \mathcal{L}''$ **do**
    Select uniformly at random one edge e from G_{seed};
    Add e to FixedCore;

$i = 1$ `// set counter`

while $i < N_s$ **do**
    $G = \emptyset$ `// empty graph`
    $G =$ FixedCore;
    while *size(G)* $<\ \mathcal{L}'$ **do**
        Select uniformly at random one edge e from Dataset$[i-1]$;
        Add e to G;
    while *size(G)* $<\ \mathcal{L}$ **do**
        Add edge to G `// according to the` G_{seed} `generating method`
    Dataset$[i] = G$;
    $i = i + 1$;

return Dataset

Supplementary Note S3.2: Characterization of the temporality of synthetic time-variyng networks

The networks generated with the algorithms proposed above can be used to understand how the presence of a core of interaction (fixed or mutating) reverberates on the average values of the temporality, $\overline{\mathcal{T}}$. Figure S1 reports the average value of the temporality computed: numerically from the synthetic data ($\overline{\mathcal{T}}$ – Data), analytically via Eq. (6) ($\overline{\mathcal{T}}_{\text{th}}$ – Theory), numerically by sampling sequences extracted from the reshuffling configuration space ($\overline{\mathcal{T}}_{\text{rand}}$, – Random), and the maximum and minimum over the reshuffling configuration space ($\overline{\mathcal{T}}_{\text{min}}$,$\overline{\mathcal{T}}_{\text{max}}$), for different synthetic datasets generated using either the FIXED CORE or the MUTATING CORE algorithm.

All the datasets have $N_s = 1500$ snapshots and $N = 500$ nodes. Then, we select two values of $\kappa = \{0.3, 0.6\}$ and three values of $\mathcal{L} = \{1500\,(\rho = 0.006),\, 2000\,(\rho = 0.008),\, 2500\,(\rho = 0.01)\}$ (where ρ is the density of edges, $\rho = \frac{2\mathcal{L}}{N\,(N-1)}$). For each (κ, ρ) pair, we generate one sequence with the FIXED CORE method and another with the MUTATING CORE one. At first glance, we notice that the average temporality of the sequence ($\overline{\mathcal{T}}$ – Data) depends almost exclusively on the percentage of persistent edges κ (it weakly depends also on ρ). The difference between both methods emerges in the relative position of $\overline{\mathcal{T}}$ and $\overline{\mathcal{T}}_{\text{th}}$, $d_{\text{th}} = \left|\overline{\mathcal{T}} - \overline{\mathcal{T}}_{\text{th}}\right|$. For those sequences with a fixed core of interactions, $d_{\text{th}} \simeq 0$. This is not the case of sequences generated through the MUTATING CORE method ($d_{\text{th}} > 0$). The non zero value of d_{th} is due to the fact that the persistence of edges between snapshots occurs only across immediately adjacent snapshots and, therefore, it is lost when performing the snapshots reshuffling process.

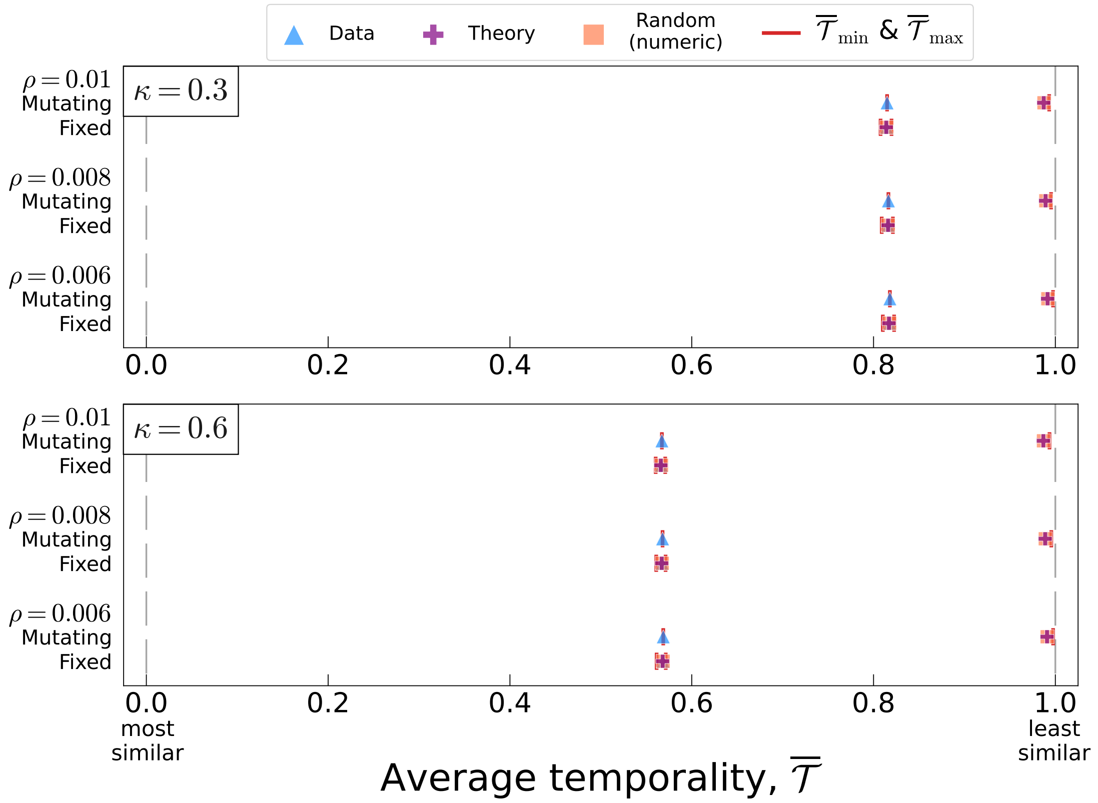

Supplementary Figure S1: Characterisation of the average temporality, $\overline{\mathcal{T}}$, of the synthetic datasets generated with the our algorithms (see Supplementary Note S3.1:). For each dataset, we display the empirical value of $\overline{\mathcal{T}}$, its theoretical estimation, the average over the reshuffling configuration space, and its maximum and minimum possible values ($\overline{\mathcal{T}}_{\text{max}}$, $\overline{\mathcal{T}}_{\text{min}}$). The vertical dashed lines at $\overline{\mathcal{T}} = 0.0$ and $\overline{\mathcal{T}} = 1.0$ highlight the temporality's theoretical boundaries.

To improve our understanding of the dependence of d_{th} on the origin of the edges' persistence, we can use synthetic sequences generated with the interpolating method and compute the value of d_{th} as a function of the parameter controlling the type of persistece, α. In Fig. S2, we show the value of d_{th} for several synthetic sequences generated with several values of α, and for two values of size of the core $\kappa = \{0.3, 0.6\}$. The values of all the other parameters are kept fixed to $N = 500$, $N_s = 1500$ and $\mathcal{L} = 1500\,(\rho = 0.006)$. As we increase α, we observe that d_{th} decreases monotonically from its maximum value (which depends on κ and is located around $1 - \overline{\mathcal{T}}$) to approximately zero as $\alpha = 1$.

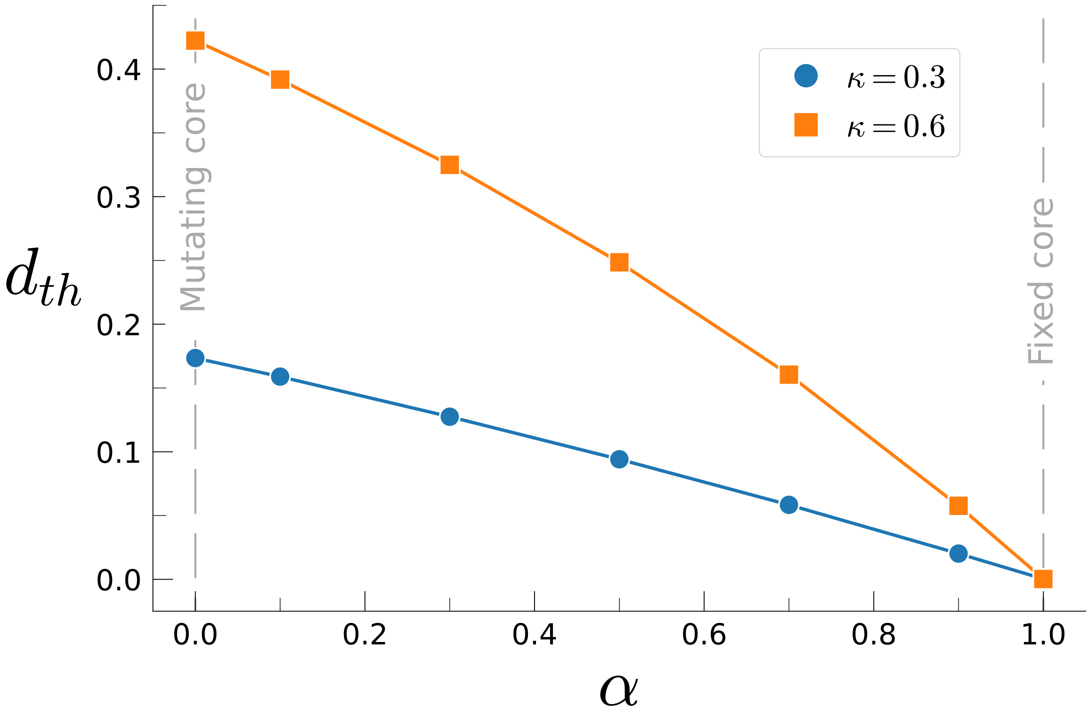

Supplementary Figure S2: Difference between the empirical and theoretical values of the temporality, d_{th}, computed for synthetic sequences generated with the interpolating method for several values of parameter α. We consider sequences with different amount of persistent edges $\kappa = 0.3$ (blue dots) and $\kappa = 0.6$ (orange squares). The values of the remaining parameters are kept fixed to $N = 500$, $N_s = 1500$ and $\mathcal{L} = 1500$ ($\rho = 0.006$). Sequences obtained for $\alpha = 0$ are equivalent to those obtained with the MUTATING CORE method, whereas sequences obtained for $\alpha = 1$ correspond to those obtained with the FIXED CORE method.

Supplementary Note S4: Extraction of trade and flights networks

We describe here the procedure we followed to generate the networks of trade and `US domestic flights`. Please check the data availability statement for information on the location of the data and the procedure used to generate them.

Trade

The trade networks can be obtained from the data collected from the United Nations (UN) COMMTRADE database [3] via its *application programming interface* or API [4]. The database stores data and statistics about official trade transaction of goods (commodities) and services between countries. Transaction records are available at either monthly or annual resolution and, depending on the commodity/country, span a time lapse from 1960 until nowadays. The structure of the data evolves over time, and oscillates between the HS format (1960–1990) and the SITC one (1990 onward). We build the time-varying networks of *export* transactions on a given commodity (`cereals`, `weapons`, and `carpets`) occurring between one country (Italy or Turkey) and all the other countries in the world (*i.e.*, they are star networks). Supplementary Table S1 summarizes the main features of each dataset. The data can be downloaded using the COMMTRADE APIs which return them encoded into the `JSON` format.

Supplementary Table S1: Resume of the trade networks' characteristics. For each network, we report the exporting country, the commodity exported, the data's format(s), the time resolution (M = monthly, Y = annual), and the time span considered.

Country	Commodity	Format	Resolution	Time-span
Italy	`cereals`<br>`weapons`	HS	M	Jan 2010 – Dec 2020
Turkey	`carpets`	HS & SITC	Y	1962 – 2020

For each data record, we extract the following meta-information:

`rt3ISO` Reporter country's ISO3 code (*e.g.*, ITA = Italy).

`pt3ISO` Partner country's ISO3 code (*e.g.*, BRA = Brazil).

`TradeValue` The trade volume (in our case, export) expressed in some unit (*e.g.*, kilograms or units) depending on the commodity.

`motDesc` Description of the mode of transportation (*e.g.*, air or sea) used to transfer the commodity. We consider the transactions made using all the transportation modes (*i.e.*, `motDesc` = `All MOTs`) available.

As the data may contain duplicate records, to avoid them, we parse only those records whose descriptors named `cstDesc` and `pt3ISO2` are equal to `All CPCs` and `W00`, respectively. Moreover, we exclude transactions towards partners whose ISO3 code corresponds to countries that no longer exist or with groups of countries (*e.g.*, ATA which stands for Antarctica).

U.S. domestic flights network

The networks are generated from the data available in the database of the U.S. Bureau of Transportation Statistics [5]. In particular, we used the information contained in the table named "T-100 Domestic Segment (U.S. Carriers)" of the "Air Carrier Statistics (Form 41 Traffic) – U.S. Carriers" database[1]. The table contains domestic non-stop segment data reported by U.S. air carriers when both origin and destination airports are located within the boundaries of the United States, operated between January 1990 and December 2021 and aggregated at a monthly resolution. The data are available as CSV files and contain several meta information. For our purposes, we considered the following fields:

`OriginAirportID` The ID of the origin airport (*e.g.*, JFK to indicate "New York, NY: John F. Kennedy International").

`DestAirportID` The ID of the destination airport.

`Freight` The amount of freight (in pounds) shipped.

`Month` The month of the year in the range $[1, 12]$ (with 1 = January and 12 = December).

Other fields include, for instance, information like `AirlineID`, `UniqueCarrierName`, or `Passengers`. We manually downloaded one file per year from 1990 until 2020 corresponding to a total of $N_s = 372$ snapshots. To build the networks, we consider exclusively those records having `Freight` ≥ 1. Finally, we store the networks as a time-stamped edge list. Both the data and the code used to extract the networks are available at [6].

[1] Available at: `https://www.transtats.bts.gov/Tables.asp?QO_VQ=EED&QO_anzr=Nv4%FDPn44vr4%FDf6n6v56vp5%FD%FLS14z%FDHE%FDg4nssvp%FM-%FD%FDh.f.%FDPn44vr45&QO_fu146_anzr=Nv4%FDPn44vr45`

Supplementary Note S5: Effects of aggregation on all the datasets

Figure S3 displays the effects of changing the temporal resolution on all the datasets considered in our study.

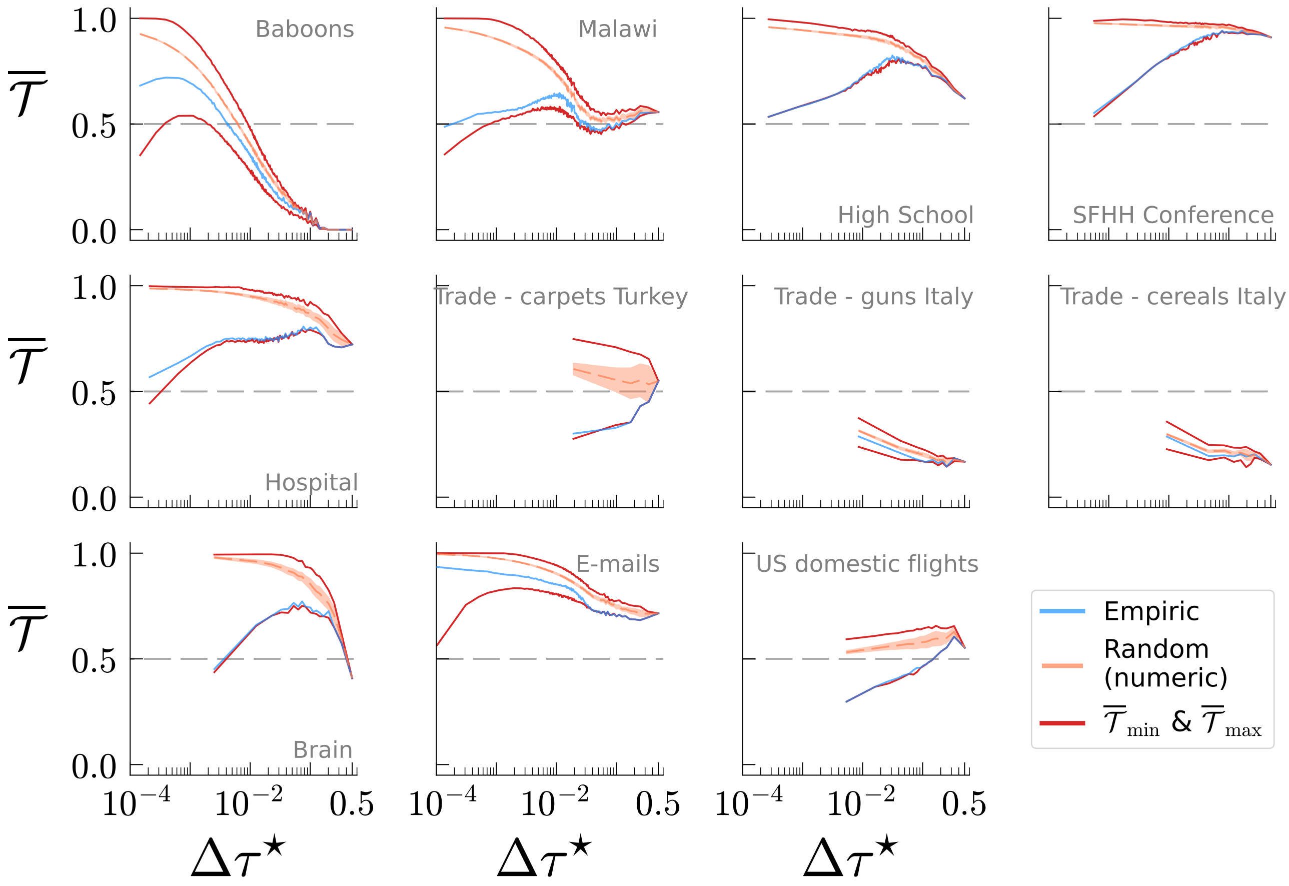

Supplementary Figure S3: Effects of the coarse graining on the temporality. Each panel refers to a distinct empiric dataset. See the caption of Fig. 3 of the main manuscript for further details and notation.

References

[1] Perra, N., Gonçalves, B., Pastor-Satorras, R. & Vespignani, A. Activity driven modeling of time varying networks. *Scientific Reports* **2**, 469, DOI: 10.1038/srep00469 (2012).

[2] Erdős, P. & Rényi, A. On random graphs. *Publicationes Mathematicae Debrecen* **6**, 290–297 (1959).

[3] United Nations COMMTRADE database. Available at: https://comtrade.un.org/ (Accessed on 01-07-2021).

[4] United Nations COMMTRADE API. Available at: https://comtrade.un.org/data/doc/api/ (Accessed on 01-07-2021).

[5] US Bureau of Transportation Statistics – TranStats. Available at: https://www.transtats.bts.gov/ (Accessed on 01-07-2021).

[6] Cardillo, A. US domestic flights datasets. Available at: https://cardillo.web.bifi.es/data.html#flights (2021).
